# Incidents of Animal Life

**Published:** 1887-02-05

**Authors:** F. O. Morris

**Affiliations:** Rector of Nunburnholme, Yorkshire; author of "British Birds," etc


### Incidents of Animal Life.

Collated by the Rev. F. O. MORRIS, B.A.,

Rector of Nunburnholme, Yorkshire; author of "British Birds," etc.

"He prayeth best, who loveth best
All things both great and small;
For the dear God who loveth us,
He made and loveth all."—COLERIDGE.

Since my anecdote last week, of the dog, I have received the two following from correspondents, both of them entire strangers to me, as so many other hundreds have been. They happen both to be very suitable for hospital patients, and the latter of them more than ordinarily so.

**A Cat Story.** "Excuse a stranger addressing you, but though no naturalist I have been so interested and instructed by your letters to THE HOSPITAL that I cannot forbear telling you what I think will interest you in the conduct of our *cats*. A tabby which we have had about thirteen years, had two and a half years ago, a son with such beautiful long soft thick hair and tail, that our man, who is very fond of animals, saved it when the rest of the brood was destroyed, no subsequent families have been preserved. The old cat asks to be let out when the servants sit down to supper at eight p.m. and soon after asks to be let in again, and then, frequently, three times last week, brings in a bird which she lays down at her son's feet and he, dutifully, accepts and eats, though he is a mouser, not afraid of a big rat and perfectly able to supply his own wants. I never met with such an instance of feline maternity, and it appears to me as worthy of record as the service of Cowper's "*Beau*" and much wanted as our example to many wretched mothers in and near the East End of London, at the present time."

**How to Feed the Birds.** "I have just read with interest your letter as to feeding the birds this severe weather. I am quite an invalid, but I thought you would like to know what a pleasure your "British Birds" is to me, and how I manage to observe them. My bed is near the window, and on the sill I have a good-sized pot, with a little oak tree (about eighteen inches high) growing in it. On the mould of the pot, I keep a constant supply of food and am much amused to find the partiality of birds for various diets. The robins like the crumb of bread the best; the tom-tits suet or scraps of meat; the chaffinches will eat farinaceous puddings; the sparrows seem omnivorous. Yesterday and to-day, I have observed a bird, which on looking carefully through your volumes, is I believe a Black Cap, and is mentioned as being a not unusual visitant in the South of Ireland. He seemed very jealous of the other birds and fought with a tom-tit and drove it away. One very pretty way is to tie one or two bacon bones, with a little meat left on, to the branch of the oak tree, then the tits will comes in great delight, and are charming to watch hopping and pecking at them. One robin is so tame he is into the room whenever the window is open, but as I have a kitten about I do not encourage this. I subjoin a list of the birds which have come to my pot this winter, but we are too much surrounded by houses to have a great variety, they have been, nevertheless, a great source of interest and pleasure to me, reminding me of Him, Who caring for the sparrows yet says to His children, "Fear not; ye are of more value than many sparrows." The great advantage of the pot is that being ten or twelve inches high it brings the birds within easy vision whereas on the sill they would be hidden by the wood work and the tree is a *great addition.* My visitors include rook, jackdaws, sparrows, chaffinches, greenfinch, robins, great tit, blue tits, cole tits, starlings, missel thrush, black cap. In the garden we have blackbirds, thrushes, and a goldfinch.

